# Different prognosis according to treatment in patients with acute promyelocytic leukemia: How the outcome changed over time

**DOI:** 10.1007/s00277-024-06014-1

**Published:** 2024-10-15

**Authors:** Emilia Scalzulli, Alessandro Costa, Ida Carmosino, Paolo Musiu, Maria Laura Bisegna, Maria Stefania De Propris, Claudia Ielo, Daniela Diverio, Clara Minotti, Saveria Capria, Roberto Latagliata, Maurizio Martelli, Massimo Breccia

**Affiliations:** 1 Hematology, Department of Translational and Precision Medicine, Az. Policlinico Umberto I- Sapienza University, Via Benevento 6, Rome, 00161 Italy; 2 Hematology Unit, Department of Medical Sciences and Public Health, Businco Hospital, University of Cagliari, Cagliari, Sardegna, Italy; 3 Hematology Unit, Belcolle Hospital, Viterbo, Italy

**Keywords:** Acute promyelocytic leukemia, ATO, ATRA, Early death, Long-term follow-up, Prognostic factors

## Abstract

A comprehensive analysis of 220 patients diagnosed with APL between 1993 and 2022 is here reported. Overall, 214 patients (97.2%) received induction therapy. Complete response (CR) was achieved in 97.4%, 100%, 100%, and 27% of patients treated with AIDA protocol, AIDA + Ara-C, ATRA + ATO, and ATRA monotherapy, respectively. Molecular complete response (CR_MRD_-) was achieved in 96.8% cases, and 142 patients proceeded to maintenance therapy. Overall, the 3-year and 5-year overall survival (OS) rates were 80.8% (95% CI, 78.1–83.5) and 79.1% (95% CI, 76.4–81.8), respectively. Considering only patients who completed induction and maintenance therapy, the 5-year OS rates were 82.1% (95% CI, 77.5–86.7) for the AIDA0493 cohort, 87.5% (95% CI, 84.4–91.1) for the AIDA2000 cohort, and 100% for the APL0406 cohort (*p* = 0.044). Additionally, the disease-free survival (DFS) rates were 65.7% (95% CI, 60.4–70.9), 70% (95% CI, 65.8–75.2), and 95.1% (95% CI, 91.7–98.5) (*p* = 0.016), respectively. Among low and intermediate-risk patients, age > 70 years (*p* = 0.027) and relapse (*p* < 0.001) were significantly associated with reduced outcomes. This study contributes to the advancement of our understanding of APL treatment, underscoring the ongoing need for research to enhance outcomes and explore new therapeutic approaches and prognostic factors.

## Introduction

Acute promyelocytic leukemia (APL) represents a significant advancement in hematology due to evolving therapeutic strategies that have substantially transformed care prospectively. Indeed, the discovery of the *PML::RARα* fusion transcript and subsequent incorporation of all-trans retinoic acid (ATRA) into treatment protocols has revolutionized APL treatment [1, 2]. The combination of ATRA and chemotherapy demonstrated remarkable outcomes, achieving high rates of complete responses (CR) and durable remissions in up to 95% of cases, along with 2-year relapse rates below 10% [3–5]. Consequently, this combined protocol has set the standard for induction therapy for each class of APL risk. Through the independent efforts of Spanish and Italian groups, the Sanz relapse risk-score enabled a risk-adapted approach, limiting intensive treatment for high-risk patients [6, 7]. The introduction of arsenic trioxide (ATO) in the 1970s has marked another milestone in APL treatment, yielding CR rates of 50–70% in relapsed/refractory patients [8] and remarkable overall survival (OS) rates of 65–75% in first-line settings [9, 10]. The established synergy between ATRA and ATO, integrated as central therapeutic components, has transformed APL treatment paradigms, providing chemotherapy-free alternatives that effectively target disease eradication [11]. Notably, the ATRA + ATO combination has proven highly effective in treating low or intermediate-risk patients, substantially reducing relapse rates and ensuring remission in a majority of cases [12]. The recent results of the APOLLO trial in high risk APL patients showed a significant event-free survival (EFS) in this setting of patients when treated firstline with ATRA-ATO compared to conventional ATRA-chemotherapy (CHT) [13].

However, APL treatment landscape is not devoid of challenges, as some issues persists in therapeutic management and risk stratification, particularly in high-risk cases. Early death (ED) remains the primary cause of treatment failure, contributing to 20–25% of initial 30-day fatalities due to fatal bleeding and thrombosis [14]. Additionally, the role of measurable residual disease (MRD) monitoring, particularly for low or intermediate-risk patients, may need revision given the markedly low relapse percentage in this patient cohort with new treatments. This study examines a cohort of adult APL patients diagnosed from 1993 to 2022. Key objectives include identifying prognostic factors related to OS at the time of diagnosis, comparing outcomes among low and intermediate-risk patients who received different treatment protocols, assessing both OS and disease-free survival (DFS) based on risk and treatment, analyzing ED and differentiation syndrome (DS) rates, and examining second-line relapses and salvage therapies. Ultimately, we seek to improve our understanding of APL and the factors influencing its course and therapeutic outcomes.

## Materials and methods

We conducted a retrospective analysis involving 220 APL patients treated at the Department of Translational and Precision Medicine, Sapienza University of Rome, from March 1993 to May 2022. Data were collected in a database, including baseline information such as age, sex, complete blood count, Sanz risk score, morphological analysis of bone marrow (BM) aspirate, molecular biology for PML-RARα transcript and isoform identification, immunophenotype, and body mass index (BMI). Morphological and molecular responses were evaluated at the end of induction and consolidation phases, respectively. Flow cytometric analysis assessed antigen expression using monoclonal antibodies, including CD34, CD15 and CD56 on BM samples. *PML::RARα* transcript was identified via qualitative PCR (qPCR), and MRD was monitored using quantitative real-time PCR (RT-PCR) following the Biomed protocol [15]. For induction response assessment, BM aspirate was collected on the twenty-eighth day and when necessary. Morphological CR (CR) was defined as restoration of normal marrow cellularity with promyelocytes count below 5%, peripheral polymorphonuclear leukocyte count > 1.5 × 10^9^/L, and platelet count > 100 × 10^9^/L. Molecular CR (CR_MRD−_) was defined as absence of the amplification band associated with the specific *PML::RARα* transcript identified at diagnosis. Molecular tests were performed on bone marrow cells after the third consolidation cycle, then every 3 months for two years and every 6 months post-maintenance. Molecular relapse was defined as the reappearance of positive RT-PCR results at any time after achieving post-consolidation CR_MRD−_, confirmed in two BM samples taken in close temporal proximity. Diagnosis of DS followed Frankel criteria [16]. As per protocols, all patients received prophylactic steroids and only high-risk patients treated according to AIDA2000 trial received prophylactic intrathecal therapy before each consolidation cycle.

Continuous variables were reported as medians and ranges, while categorical variables as frequencies and percentages. The Wilcoxon-Mann-Whitney test was used for non-parametric series comparison, and Fisher’s exact test for categorical comparison. A significance level of *p* < 0.05 was considered statistically significant. OS was measured from diagnosis to patient’s death or last follow-up visit and calculated using the Kaplan-Meier method. DFS was measured from CR achievement to relapse or death from any cause, censoring living patients without evidence of relapse at last contact. OS and DFS were estimated using Kaplan-Meier and compared with the long-rank test. In multivariate analyses, the Cox proportional hazards model was used for DFS and OS. Cumulative incidence of relapse (CIR) was defined as the time from the end of induction therapy to the date of hematologic, molecular, or extramedullary relapse, whichever occurred first, considering death in remission as a competing event. The Gray test was used to assess differences in CIR between groups defined by patient characteristics. Statistical analysis was performed using IBM SPSS Statistics software. The study was conducted according to declaration of Helsinki and after obtained informed consent by all patients.

## Results

### Patients’ cohort

The analysis was conducted on 220 APL patients. Patient characteristics are summarized in Table 1. At diagnosis, patients had a mean age of 50 years (range: 19–85 years), with 49% being male. Median values of hemoglobin, leukocytes, and platelets at diagnosis were 9.9 g/dL (range: 3.2–15 g/dL), 2.3 × 10^9^/L (range: 0.4–286 × 10^9^/L), and 29 × 10^9^/L (range: 2-302 × 10^9^/L), respectively. Classic morphological phenotype was observed in 78% of cases. The molecular transcript type of *PML::RARα* at diagnosis was bcr1-2 in 54% of patients and bcr3 in the remaining 46%. According to the relapse risk score, patients were classified as low risk in 29% (*n* = 64), intermediate risk in 45% (*n* = 98), and high risk in 26% (*n* = 58). Among 199 evaluable baseline patients, 17 were CD15^+^ (8.5%), 61 were CD34^+^ (30.6%), and 21 were CD56^+^ (10.5%). CD34 expression was more frequently associated with the morphological variant (*p* < 0.001), high risk (*p* < 0.001), and elevated leukocyte count at onset (*p* < 0.001). Conversely, CD15 antigen positivity correlated with classic morphology (*p* = 0.018), while CD56 expression was associated with the bcr3 transcript (*p* = 0.002). Regarding BMI assessment, 57% of the 156 evaluable patients had a BMI > 25.

**Table 1:** Patients’ characteristics at diagnosis

Characteristics	Total cohort (*n* = 220)
Age, median years (range)	50 (19–85)
Males, n (%)	108 (49)
White Blood Cells (x10^9^/L), median (range)	2.3 (0.4–280)
Platelets (x10^9^/L), median (range)	29 (2-302)
Hemoglobin (g/dL), median (range)	9.9 (3.2–15)
Morphology, M3/M3v; n (%)	171/49 (78/22)
Immunophenotype	
CD15+	17 (8.5)
CD34+	61 (30.7)
CD56+	21 (13.1)
*PML::RARα*, n (%)	
Bcr1	111 (50.7)
Bcr2	9 (4.1)
Bcr3	99 (45.2)
Sanz risk score, n (%)	
Low	64 (29.1)
Intermediate	98 (44.5)
High	58 (26.4)
Early death risk score, n (%)	
Low	111 (50.7)
Hight	79 (35.9)
Very High	30 (13.6)

### Induction therapy and differentiation syndrome

Overall, 214 patients (97.2%) underwent induction treatment, and 202 (94.3%) were assessable for response at completion. Treatment regimens included AIDA in 158 patients (73.8%), AIDA in combination with cytarabine (Ara-C) in seven cases (3.3%), ATO + ATRA combination in 38 cases (17.8%), and ATRA monotherapy in 11 cases (5.1%). CR post-induction was achieved in 154/158 (97.4%), 7/7 (100%), 38/38 (100%), and 3/11 (27%) of these groups, respectively. The median time to achieve CR was 37.5 days (SD: ± 20.05).

DS occurred in 42/214 patients (19.6%), including 27 cases among AIDA-treated patients (17%), 4 cases of AIDA + Ara-C (57%), 6 cases of ATRA + ATO (23%), and 2 cases of ATRA monotherapy (18%). Based on risk classification, DS occurred more frequently in high-risk (36.3%) than intermediate-risk (19.7%) or low-risk (4.7%) patients. Only one case was fatal due to respiratory failure. Univariate statistical analysis demonstrated a significant association between elevated leukocyte count at diagnosis and DS (*p* = 0.003).

Sixteen deaths (7.2%) were recorded during the early disease phase (i.e., 0–30 days from diagnosis), including 4 before induction therapy initiation and 12 during or after induction. The median age of these subjects was 65 years (range: 23–85), median leukocyte count was 15.3 × 10^9^/L (range: 0.7–286 × 10^9^/L), platelet count was 29 × 10^9^/L (range: 14–116 × 10^9^/L), and hemoglobin was 8.2 g/dL (range: 3.8–14.8 g/dL). None of these patients belonged to the low-risk category, while 6 were intermediate-risk, and 10 were high-risk. Overall, causes of death included cerebral hemorrhage (*n* = 3) and cerebral ischemia (*n* = 1) in patients who died before treatment initiation, while hemorrhagic complications (cerebral hemorrhage, *n* = 7; gastrointestinal bleeding, *n* = 1), infections (sepsis, *n* = 3), and disseminated intravascular coagulation (DIC, *n* = 1) were causes of death in the 12 patients who died during or after induction therapy.

### Consolidation therapy

Among the 202 patients achieving CR after induction, 196 underwent consolidation therapy. Protocols and outcomes are shown in Table 2. Three patients died post-induction and prior to consolidation, while an additional three, due to low performance status, skipped consolidation and proceeded directly to maintenance therapy. Within the 196 patients, treatment distribution was as follows: 67 patients (34.1%) received chemotherapy according to AIDA0493, 85 (43.3%) followed AIDA2000 protocols, 39 (19.8%) received ATRA + ATO therapy, three patients (1.5%) received gemtuzumab ozogamicin (GO) monotherapy, one patient (0.6%) received idarubicin monotherapy, and another one (0.6%) received ATRA monotherapy.

**Table 2:** Treatment protocols and outcomes of APL patients who received salvage therapy

Cohorts	5-year OS, % (95% IC)	*p*	5-year DFS, % (95% IC)	*p*
*Based on risk category*				
Low risk	90.5 (75.2–86.3)	**0.004**	77.8 (72.7–82.9)	**0.324**
Intermediate risk	78.9 (74.7–84.1)		70.8 (66.1–75.6)	
High risk	66.1 (59.9–72.3)		67.4 (60.6–74.2)	
*Based on treatment protocols*†				
AIDA0493	82.1 (77.5–86.7)	**0.044**	69.6 (60.4-70-9)	**0.16**
AIDA2000	87.5 (84.4–91.1)		70 (65.8–75.2)	
APL0406	100		95.1 (91.7–98.5)	
*Low- and intermediate risk cohort*				
AIDA0493	83.3 (77.8–88.2)	**0.049**	64.8 (59.8–70.1)	**0.022**
AIDA2000	90 (82.5–93.9)		72 (65.9–78.5)	
APL0406	100		94.9 (91.3–98.6)	

† Patients who completed induction and consolidation therapy

Out of the patients evaluable for response (*n* = 191), CR_MRD−_ was achieved in 96.8%. Among the remaining six patients, five patients died to treatment-related complications, while one patient did not achieve CR_MRD−_. The average time to attain CR_MRD−_ was 156.7 days (SD: ± 70.15), with a median of 160 days (interquartile range: 101-188.5).

### Maintenance therapy

After achieving CR_MRD−_, 142 patients proceeded to maintenance therapy. Among these, 66.2% received a combination of ATRA, methotrexate (MTX), and 6-mercaptopurine (6-MP), 24.7% received ATRA monotherapy, 7.7% received MTX and 6-MP, and 1.4% received ATRA + interferon. The mean duration of maintenance therapy was 22 months (SD: ± 7), with a median of 25.3 months (interquartile range: 22.8–27.1). During the maintenance phase, recurrence was observed in 21 patients: 11 patients were undergoing combination therapy of ATRA, MTX, and 6-MP, five were on MTX and 6-MP regimen, and five were receiving ATRA monotherapy.

### Outcome and survival

Overall, the 3-year and 5-year OS were 80.8% (95% CI, 78.1–83.5) and 79.1% (95% CI, 76.4–81.8), respectively. Stratifying patients by risk groups, the 5-year OS were 90.5% (95% CI, 86.8–94.1) for low-risk, 78.9% (95% CI, 74.7–84.1) for intermediate-risk, and 66.1% (95% CI, 59.9–72.3) for high-risk patients (*p* = 0.004).

**Fig. 1:**
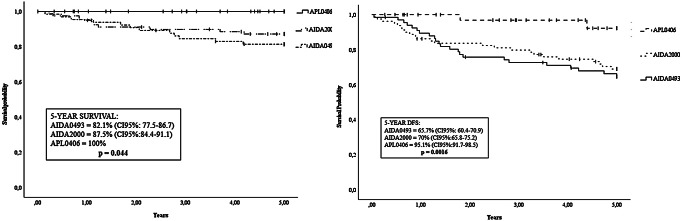
Overall Survival (OS) and Disease-Free Survival (DFS) based on treatment protocol

Considering only patients who completed induction and maintenance therapy according to AIDA0493, AIDA2000, and APL0406 protocols, the 5-year OS was 88% (95% CI, 85.1–91.3) while the 5-year DFS was 73.9% (95% CI, 70.5–77.2). Stratifying by protocol (Fig. 1), the 5-year OS were 82.1% (95% CI, 77.5–86.7) for the AIDA0493 cohort, 87.5% (95% CI, 84.4–91.1) for the AIDA2000 cohort, and 100% for the APL0406 cohort (*p* = 0.044), while DFS (Fig. 1) was 65.7% (95% CI, 60.4–70.9), 70% (95% CI, 65.8–75.2), and 95.1% (95% CI, 91.7–98.5) (*p* = 0.016), respectively.

**Fig. 2:**
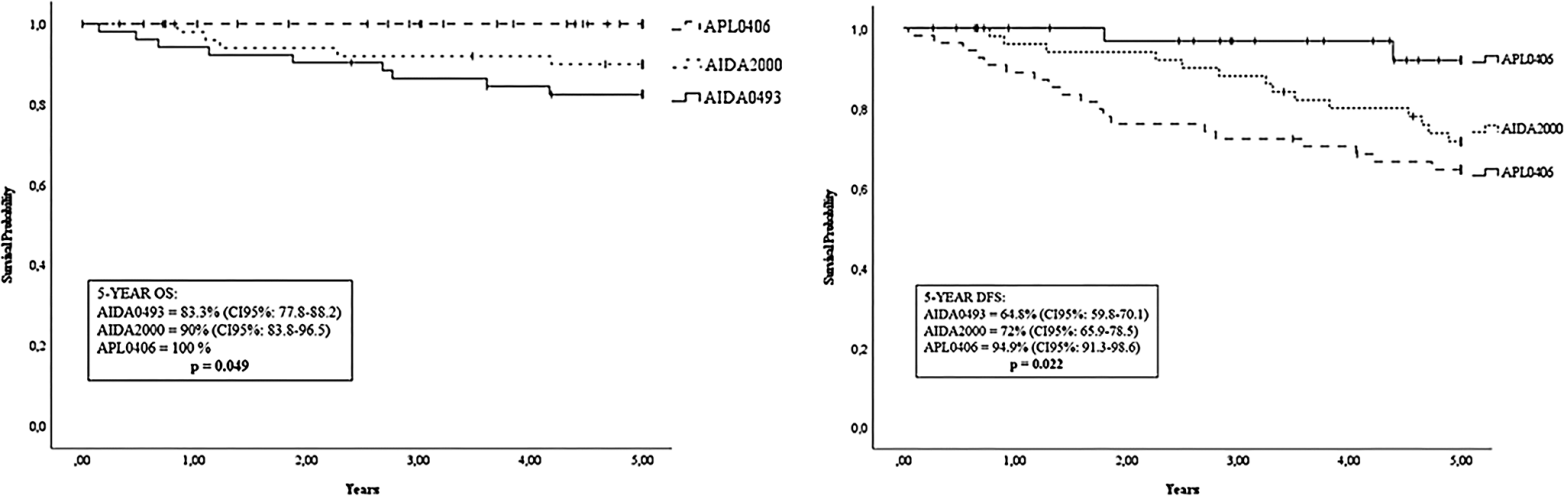
5-year Overall Survival (OS) and Disease-Free Survival (DSF) of low and intermediate-risk patients who completed induction and consolidation treatment according to conventional protocols

In low and intermediate-risk patients the 5-year OS based on received treatment was 83.3% (95% CI, 77.8–88.2) for AIDA0493, 90% (95% CI, 83.8–96.5) for AIDA2000, and 100% for APL0406 protocols (*p* = 0.049) (Fig. 2). The 5-years DFS in these patients was 64.8% (95% CI, 59.8–70.1), 72% (95% CI, 65.9–78.5), and 94.9% (95% CI, 91.3–98.6) for AIDA0493, AIDA2000, and APL0406 protocols, respectively (*p* = 0.022) (Fig. 2).

**Fig. 3:**
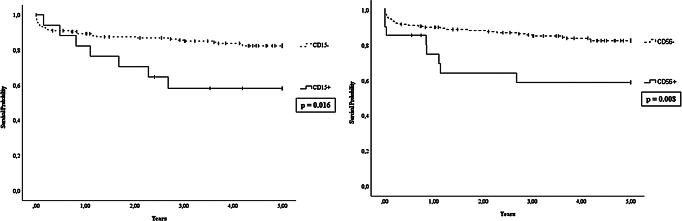
5-Year Overall Survival (OS) based on the expression of CD15 and CD56 antigens

Furthermore, a statistically significant reduction in 5-year OS was observed in patients with CD15^+^ APL (58.8% vs. 83.2%, *p* = 0.016) and CD56^+^ APL (62.9% vs. 83.4%, *p* = 0.008), while the 5-year DFS was 90.2% (95% CI, 87.8–92.6) and 75.5% (95% CI, 71.9–79.1), respectively (Fig. 3).

No differences were observed in terms of 5-year OS (81% vs. 89.9%, *p* = 0.963) or DFS (78% vs. 71.4%, *p* = 0.526) among patients who developed DS. In total, 61 deaths were recorded. Among these, 19 occurred at diagnosis and during induction therapy, as discussed earlier. From univariate analysis, increased risk at diagnosis (*p* = 0.001) and age over 70 years (*p* = 0.013) were associated with an elevated risk of ED, maintaining significance in multivariate analysis (*p* = 0.001 and *p* = 0.024). Among the remaining 42 episodes, the main causes were disease progression in 19 cases (45.2%), cerebral hemorrhage in 6 cases (14.3%), therapy-related acute myeloid leukemia (tr-AML) in 5 cases (11.9%), decompensated cirrhosis and complications after allogeneic transplantation in 2 cases each (4.7% each), SARS-CoV-2 pneumonia, gastrointestinal bleeding, secondary neoplasm, pneumonia, syncope, sepsis, therapy-related myelodysplastic syndrome, and cardiac disease in one case each (2.4% each).

Overall, clinical characteristics associated with 5-year OS in univariate analysis included female gender (85.7% vs. 73%, *p* = 0.018), age < 70 years (82.41% vs. 59.3%, *p* < 0.001), and no relapse (94.7% vs. 56.3%, *p* < 0.001). In multivariate analysis, only age < 70 years (*p* = 0.016) and absence of relapse (*p* = 0.001) remained significantly correlated. Among low and intermediate-risk patients who completed induction and consolidation therapy, 5-year OS was improved in patients aged > 70 years (85.8% vs. 64.7%, *p* = 0.027) and those without relapse (94.9% vs. 57.5%, *p* < 0.001). In multivariate analysis, APL0406 protocol therapy (*p* < 0.001) and absence of relapse (*p* < 0.001) maintained statistically significant correlation. Additionally, no benefit was observed from maintenance therapy in terms of 5-year OS (87.9% vs. 95.2%, *p* = 0.241) in these cohort.

### Relapse

Overall, 48 relapses were reported, including 10 extramedullary relapses (9 in the central nervous system and 1 cutaneous). Considering evaluable patients, the 5-year CIR was 24.4% (95% CI, 20.8–28.1). Stratifying patients by risk groups, CIR in low-risk patients was 14.3% (95% CI, 8.9–17.4), in intermediate-risk group was 26.7% (95% CI, 22.1–30.3), and in high-risk patients was 29.2% (95% CI, 22.6–35.8), with no statistically significant differences among the three groups (*p* = 0.097).

**Fig. 4:**
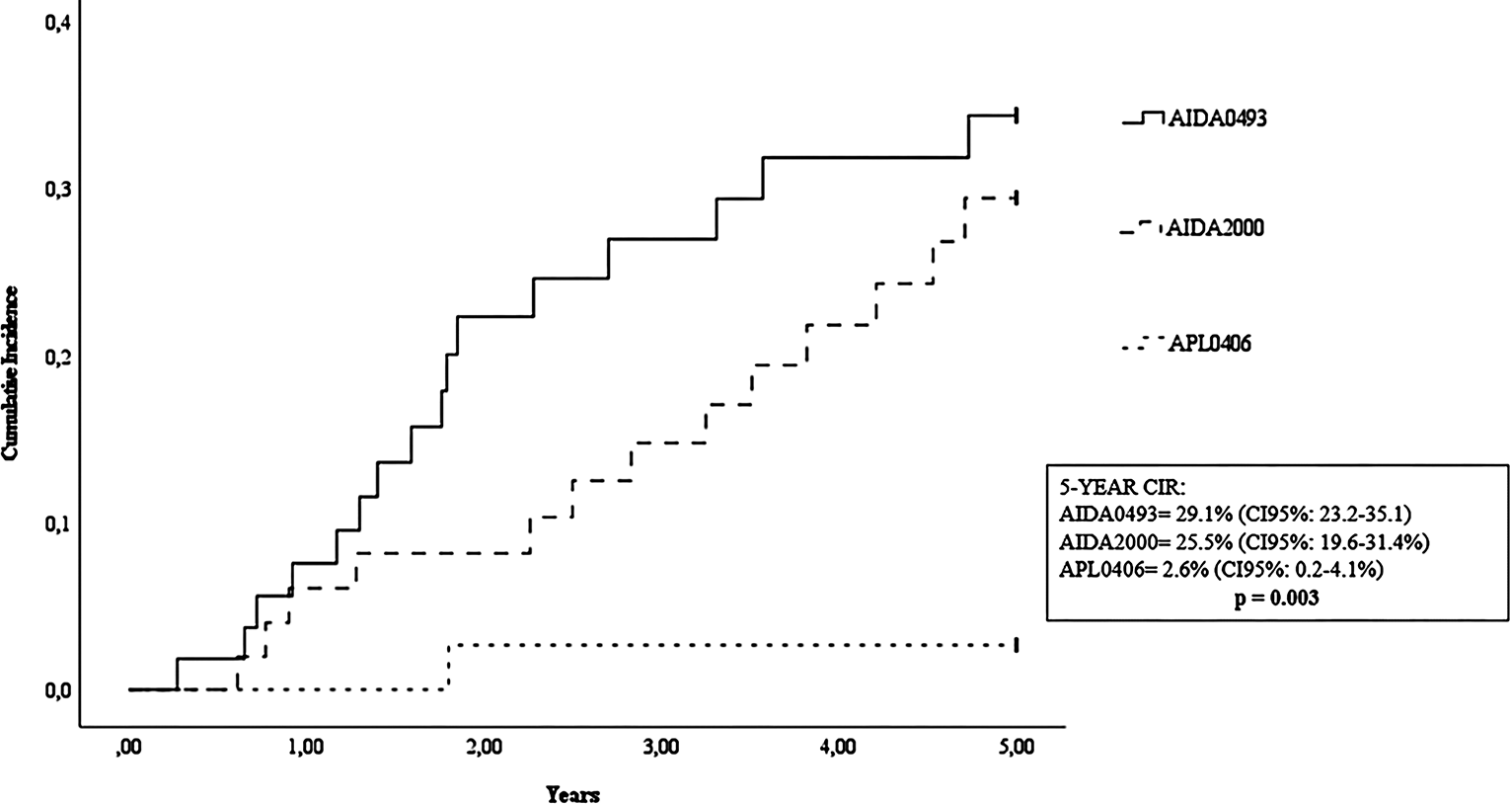
Cumulative Incidence of Relapse (CIR) at 5 years in Intermediate and low-risk patients following Induction and consolidation therapy

Among patients who completed induction and consolidation therapy, CIR was 31.9% (95% CI, 26.6–36.2) for those treated with AIDA0493, 25.3% (95% CI, 20.4–30.2) for AIDA2000, and 2.5% (95% CI, 0.2–4.8) for APL0406 (*p* = 0.001) (Fig. 4). Only one relapse was recorded in the ATRA + ATO cohort.

Statistical analysis showed a significant difference in CIR among APL CD56^+^ cases (38.8% vs. 19.1%, *p* = 0.028), with a near-significant trend observed for patients with variant morphology compared to classical morphology (34.1% vs. 20.2%, *p* = 0.055).

### Salvage therapy

Among the 48 relapsed cases, 42 (87.5%) received salvage therapy, while five patients (10.4%) died due to disease progression and one patient (2.1%) was lost to follow-up after relapse. Details of salvage therapies in first-relapse patients are summarized in Table 3. Overall, 13 patients (30.9%) received ATRA + polychemotherapy, 10 (23.9%) received ATRA + ATO, 9 (21.4%) underwent ATRA + polychemotherapy followed by autologous hematopoietic stem cell transplantation (auto-HSCT), 4 (9.6%) received GO + ATRA, 3 (7.1%) patients were treated with ATO monotherapy, 2 patients (4.7%) received ATRA monotherapy or in combination with ATO followed by auto-HSC, and 1 patient (2.4%) with allogeneic HSCT.

**Table 3:** Salvage therapies in patients at first relapse, treatment, and outcomes

Salvage therapy		CR	CR_MRD−_
	**n = 42**	**n**,** %**	**n**,** %**
ATRA + ARA-C + MITOX	10	3 (30%)	7 (70%)
ATRA + ARA-C + IDA + GO	1	1 (100%)	-
ATRA + ATO + MTX + PL + RT	1	-	1 (100%)
ARA-C + MITOX + ATRA + IFN	1	-	1 (100%)
ATRA + ATO	10	3 (30%)	7 (70%)
ATRA + GO	4	-	3 (75%)
ATO	3	-	1 (33.3%)
ATRA + IFN	1	1 (100%)	-
ATRA + ARA-C + MITOX + auto-HSCT	9	1 (11.1%)	8 (88.9%)
ATRA ± ATO + auto-HSCT	2	-	2 (100%)

ARA-C, Cytarabine; ATO, Arsenic Trioxide; ATRA, All-trans Retinoic Acid; HSCT, Hematopoietic Stem Cell Transplant; IDA, Idarubicin; IFN, Interferon; MITOX, Mitoxantrone

Among the relapsed patients, 31 (64.5%) have died. Among the treated patients, 9 (21.4%) achieved a second CR, and 30 (71.4%) a second CR_MRD−_, while 3 (7.1%) cases were refractory to treatment.

Out of the 9 patients who achieved a second CR, 7 died, and 2 experienced a second morphological relapse, treated by allogeneic HSCT. Among the deceased patients, 5 died due to disease progression, 1 due to tr-AML, and 1 patient due to cardiotoxicity after a second relapse treated with allogeneic HSCT.

Among the 30 patients achieving a second CR_MRD−_, 14 sustained a durable response, with 10 alive at last follow-up (4 treated with ATRA + ATO, 4 with auto-HSCT after ATRA + polychemotherapy, and 1 with ATRA + ATO followed by auto-HSCT). Four patients died due to tr-AML, complications post auto-HSCT, complicated liver cirrhosis, and SARS-CoV-2 pneumonia.

The remaining 16 patients had subsequent disease relapse, with 11 undergoing third-line treatment, including ATRA + ATO (*n* = 2), ATO monotherapy (*n* = 2), GO (*n* = 1), ATRA + ATO + GO (*n* = 1), and allogeneic HSCT (*n* = 4). Moreover, three patients refractory to second-line therapy received salvage treatment, 2 with ATO monotherapy and 1 with ATRA + GO. Statistical analysis showed a reduced incidence of second relapse in patients treated with auto-HSCT in the second CR (36.4% vs. 70.9%, *p* = 0.049), without significant impact on OS rates (35.5% vs. 45.4%, *p* = 0.407).

## Discussion

This retrospective study examined 220 APL patients diagnosed between 1993 and 2022. The extended follow-up period allows the analysis of late complications, particularly in the setting of patients treated with intensive treatments, although limited by its retrospective nature and lack of randomization. Regarding epidemiological characteristics, median age, gender distinctions, cell counts, risk class, morphology, and *PML::RARα* transcript closely resembled those reported in major international trials [7, 12, 17–21]. 

Overall, the rate of EDs observed in our study (7.2%), primarily linked to disease-associated coagulopathy, was found to be in line with major international trials but lower than reported in similar retrospective studies conducted during the same period [14, 22, 23]. This difference may be attributed to the presence of a specialized Emergency Department for hematological diseases in our institution with consequent early diagnosis. Comprehensive medical education, especially in emergency departments, regarding prompt diagnostic recognition, appropriate early management with transfusion support therapy, and the rapid initiation of ATRA upon suspicion of APL diagnosis, appear to be crucial in APL management [24, 25]. 

In our cohort, the 5-year OS rates within different risk classes at diagnosis (90.5%, 78.9%, and 66.1% in the low, intermediate, and high-risk cohorts) reaffirm the predictive value of the Sanz score (Table 2). Conversely, no differences emerged in terms of 5-year DFS (77.8%, 70.8%, and 67.4%, *p* = 0.324), which may be related with patients who experienced ED in the intermediate and high-risk groups, who are excluded from DFS analysis by definition.

When comparing patients who completed the AIDA0493, AIDA2000, and APL0406 protocols for induction, consolidation, and maintenance (if applicable), the APL0406 protocol showed a significant advantage in terms of 5-year OS with rates of 82.1%, 87.5%, and 100%, respectively (*p* = 0.044). However, it’s important to note that the APL0406 protocol exclusively enrolled low and intermediate-risk patients and was introduced more recently compared to the other two protocols.

When considering only low and intermediate-risk patients, the advantage remained evident, both in terms of OS (AIDA0493 83.3%, AIDA2000 90%, APL0406 100%, *p* = 0.049) and DFS (AIDA0493 64.4%, AIDA2000 72%, APL0406 94.4%, *p* = 0.022) (Fig. 2**).** These results reflect the significant therapeutic success achieved in low and intermediate-risk patients with the combination of ATRA and ATO in both induction and consolidation therapy, consistent with the final results of the APL0406 trial, which showed, with a median follow-up of 40.6 months, OS and DFS rates of 99.2% and 97.3%, respectively [12]. Furthermore, this aligns with another recent real-life experience in which the one-year OS of 154 APL patients at low and intermediate Sanz risk treated with ATO + ATRA was 97% (95% CI, 94.100%).[26]

Another notable aspect is the low mortality associated to DS, observed in 19.6% of the entire cohort, mainly in the intermediate and high-risk cohorts (93%), with only one fatality (2.4%). This outcome is likely attributed to prophylactic steroid administration, early recognition, efficient support, and cytoreductive therapy in hyper leukocytic patients, minimizing adverse events and associated mortality.

In our study, we observed a positive impact on survival among patients aged over 70 years and those of the female gender. Specifically, we documented 27 patients (12.2%) aged over 70 years, with a 5-year OS rate of 44% and an ED rate of 20%. These results are in accordance with prior research, consistently indicating poorer outcomes in this age group [26]. This discrepancy is attributed to the heightened coagulopathy at disease onset and the higher prevalence of cardiovascular diseases and organ dysfunction, which contribute to increased inherent frailty and reduced treatment tolerance in elderly patients [27–29]. A recent international cooperative trial focused on APL patients aged over 70 years and compared outcomes between those treated with the combination of ATO + ATRA with or without cyclophosphamide, and a control arm treated with solely ATRA and cyclophosphamide [30]. The experimental arm showed superior CR rates and a reduced CIR, with significant benefits in high-risk patients. Consequently, ATO + ATRA combination therapy is becoming an essential component of treatment in this setting, regardless of their Sanz risk classification. In our study, although the sample size is limited, we treated five low and intermediate-risk patients aged over 70 years according to the APL0406 protocol. These patients achieved CR after induction and CR_MOL_ after consolidation, indicating their favorable response to the therapy and overall good tolerance.

In our immunophenotypic analysis, a significant difference was found in outcomes linked to specific antigens not typically expressed by APL blasts. The CD56 antigen, usually found in hematopoietic stem cells and indicative of increased proliferation, was detected in 21 out of 160 patients (13.1%). This positivity was associated with the bcr3 transcript and identified a subgroup of patients with a lower 5-year OS rate (Fig. 3) and higher relapse rate. These findings are consistent with previous research linking CD56 antigen positivity to higher leukocyte counts at onset, the presence of the bcr3 transcript, and notably elevated relapse rates, particularly in extramedullary sites. Therefore, CD56 expression seems to be a critical independent prognostic factor in assessing and treating patients with this hematological condition [31, 32]. However, it should be noted that the majority of patients in our study, as well as those in existing literature, received treatment regimens that included chemotherapy combined with ATRA. Therefore, the prognostic significance of CD56 positivity still requires validation in the population treated with protocols involving the ATO + ATRA combination without chemotherapy. In our cohort, we also observed CD15 in 8.5% of patients. This finding was significantly linked with lower OS rates and the classical morphology. This results are consistent with previous experiences at our center [33], while other studies have linked CD15 expression to an increased risk of arterial and venous events [34]. 

The absence of relapse was correlated with improved OS, both in univariate and multivariate analyses (*p* < 0.001). This highlights the critical role of effective first-line therapies, particularly in preventing relapse. Focusing on low and intermediate-risk patients, APL0406 protocol also showed improved OS in multivariate analysis, confirming the effectiveness and safety of ATO + ATRA in this context. Incorporating ATO into induction and consolidation therapy, even in high-risk patients, may represent a significant therapeutic avenue, despite some conflicting results. Notably, the inclusion of ATO in induction and consolidation therapy alongside ATRA + chemotherapy (idarubicin or daunorubicin) for high-risk APL patients compared to the non-ATO arm with additional cytarabine doses, did not yield statistically significant differences in OS, DFS and relapse incidence, which were notably high in both groups (ATO vs. non-ATO: 94.5% vs. 95.3%; 93.2% vs. 87.4%; 5.1% vs. 9.9%).[35] The efficacy and safety of the ATO + ATRA + GO combination were assessed in the phase II SWOG 0535 trial for high-risk patients, with minimal use of chemotherapy. This approach yielded impressive 3-year OS and DFS rates of 86% and 76%, respectively [36]. Currently, the multicentric European APOLLO trial (NCT02688140) is evaluating the integration of ATO into induction and consolidation therapy for high-risk APL, and its results are eagerly anticipated.

In terms of salvage therapy, our cohort showed a significant advantage in terms of second relapse occurrence with consolidation via auto-HSCT. However, this advantage did not translate into improved OS. Specifically, 11 patients underwent auto-HSCT, with five achieving long-term molecular remission, three experiencing a second relapse followed by death, and three others died for treatment-related toxicity. It’s noteworthy that two of the latter patients belonged to the low-risk cohort initially treated with the AIDA0493 protocol, which included chemotherapy. Based on the available literature, the role of auto-HSCT in achieving a second molecular remission remains a subject of debate. Nevertheless, several studies suggests better OS and DFS for patients who undergo auto-HSCT compared to those who do not [37–39]. 

In summary, this study confirms the efficacy of ATO + ATRA therapy for low and intermediate-risk APL patients, achieving sustained remissions with minimal short-term and long-term toxicity. Recent international protocols suggest its potential use for high-risk patients with reduced chemotherapy dosage, improving outcomes compared to older regimens with higher mortality and relapse rates. The role of autologous transplantation in second molecular remission consolidation is partially confirmed, but its effectiveness depends on frontline chemotherapy doses. Further research is needed to evaluate outcomes with chemotherapy-free or low-dose regimens. Age and Sanz risk at diagnosis remain significant prognostic factors, and CD15 and CD56 antigens may also be relevant, pending validation in patients treated with modern schemes. In conclusion, this study advances APL treatment understanding, emphasizing the need for ongoing research to improve outcomes and explore new therapeutic approaches and prognostic factors.

## Data Availability

No datasets were generated or analysed during the current study.
